# Investor attention and cryptocurrency: Evidence from the Bitcoin market

**DOI:** 10.1371/journal.pone.0246331

**Published:** 2021-02-01

**Authors:** Panpan Zhu, Xing Zhang, You Wu, Hao Zheng, Yinpeng Zhang

**Affiliations:** 1 School of Mathematics and Finance, Chuzhou University, Chuzhou, Anhui, China; 2 The School of Finance, Renmin University of China, Beijing, Beijing, China; 3 School of Economics, Beijing Technology and Business University, Beijing, Beijing, China; 4 China Youth & Children Research Center, Beijing, Beijing, China; 5 College of Economics, Shenzhen University, Shenzhen, Guangdong, China; University of Almeria, SPAIN

## Abstract

This paper adds to the growing literature of cryptocurrency and behavioral finance. Specifically, we investigate the relationships between the novel investor attention and financial characteristics of Bitcoin, i.e., return and realized volatility, which are the two most important characteristics of one certain asset. Our empirical results show supports in the behavior finance area and argue that investor attention is the granger cause to changes in Bitcoin market both in return and realized volatility. Moreover, we make in-depth investigations by exploring the linear and non-linear connections of investor attention on Bitcoin. The results indeed demonstrate that investor attention shows sophisticated impacts on return and realized volatility of Bitcoin. Furthermore, we conduct one basic and several long horizons out-of-sample forecasts to explore the predictive ability of investor attention. The results show that compared with the traditional historical average benchmark model in forecasting technologies, investor attention improves prediction accuracy in Bitcoin return. Finally, we build economic portfolios based on investor attention and argue that investor attention can further generate significant economic values. To sum up, investor attention is a non-negligible pricing factor for Bitcoin asset.

## Introduction

Currently, cryptocurrency, i.e., Bitcoin, raises great concerns with the help of promotion in technology [1–4]. As an alternative payment method accepted by merchants, i.e., Subway and Microsoft, Bitcoin is playing an increasingly important role in cryptocurrency exchanges around the world. The novelty of Bitcoin and other cryptocurrencies, as well as Bitcoin’s unprecedented performance and volatility since its inception, have drawn attention from practitioners, regulators, and scholars [5–8].

Bitcoin has received great concerns in the academic area since 2008 [9]. However, Bitcoin was traded at a low price and trading volume before 2017. At the end of 2017, Chicago Board Options Exchanges (CBOE) began to trade the Bitcoin futures [10], which made the price and trading volume of Bitcoin to raise sharply, resulting the number of institutions supporting Bitcoin payments gradually increased. In 2018, the price of Bitcoin decreased rapidly and later, the price raised slightly in 2019. The drastic changes in Bitcoin price indicate a fact that there is no difference between Bitcoin market and the general financial markets.

Return, as well as volatility, play important roles in numerous financial aspects, e.g., asset pricing, investment portfolio allocation and risk management, etc., and are the two most important characteristics of one certain asset. However, there are still many puzzles needed to be solved urgently in explaining and forecasting the Bitcoin market, which attracted numerous researchers in this field [3, 6, 11]. Investor attention, which may be represented by extreme return, abnormal trading volume, advertising expenditure, and media coverage [12–14], is a key resource constrained by limited processing capacity and time pressure, besides, it is a scarce resource for every asset, as investors can only concentrate on limited set information in reality since their time and effort constraint [13, 15, 16]. In fact, investor attention had been applied in traditional financial markets, i.e., stock market and FX market, and proved to be an influential factor in certain markets. In this paper, we provide new empirical evidence on the novel factor, i.e., investor attention, and argue that the new variable can be used for explaining and forecasting the Bitcoin market.

In this paper, we combine the cryptocurrency market and behavioral finance by making comprehensive investigations on the sophisticated relationships between Bitcoin market and investor attention. Specifically, we focus on the relationships between investor attention and the two most important characteristics of one certain asset, i.e., returns and realized volatility, and provide more empirical evidence to support that investor attention is a non-negligible factor in Bitcoin market. To the best of our knowledge, this paper makes the following contributions to the existing literature. First, we implement the basic linear granger causality tests and the corresponding VAR models to certify the linear relationships between Bitcoin market and investor attention. The results indicate that investor attention is surely the granger cause for both Bitcoin return and realized volatility. Besides, the impulse response from VAR models showed that shock from investor attention may last for several weeks in Bitcoin market. The empirical results may shed lights on investors in Bitcoin market to focus more on the variations in behavioral variable; Second, existing studies mainly focused on the linear connections between Bitcoin market and investor attention, failing to comprehensively explore the non-linear connections between the two. Therefore, current research may be incomplete in explaining the relationships between investor attention and Bitcoin market. In this paper, we fill this potential gap by four non-linear specifications adopted by previous studies in other financial area. The results certified the existence of non-linear connections between investor attention and Bitcoin market both in return and realized volatility. The empirical results also remind investors to focus both on linear and non-linear connections when analyzing Bitcoin market and investor attention; Third, we implement several out-of-sample predictions of Bitcoin return and realized volatility based on investor attention, as current research was less involved in the field. The basic one period ahead prediction regarding the Bitcoin return shows that predictive models significantly outperform the benchmark model, i.e., the historical average benchmark model. Furthermore, we do several long horizon predictions in 2 and 4 weeks to further explore the predictive power of investor attention. The results show supports to include investor attention in forecasting models regarding the Bitcoin return. However, predictive models do not outperform the benchmark historical average regarding the realized volatility for both short-term and long-term predictions. The results for out-of-sample predictions further illustrate the importance of investor attention in Bitcoin market and will surely guide the investors to forecast the Bitcoin return with the investor attention. Moreover, the empirical results add evidence on the in-sample and out-of-sample analysis; Fourth, based on the empirical results of out-of-sample predictions for Bitcoin return, we construct several simple portfolios including Bitcoin asset and risk-free asset to further explore the usefulness of investor attention in Bitcoin portfolio management based on the framework of asset allocation. Specifically, we compare the performance of several benchmark models and several predictive models, and the results show that predictive models incorporated with investor attention surely improve the sharp ratio. The results provide important suggestions for investors, as investor attention can further generate significant economic values.

The rest of this paper is organized as follows. Section 2 briefly reviews the relevant literature of the Bitcoin market and investor attention. Section 3 introduces the data used in this paper. Empirical results for in-sample estimations and out-of-sample predictions are shown in Section 4 and Section 5, respectively. In Section 6, we construct portfolios and the related results are shown in this part. Section 7 concludes the paper.

## Literature review

Since Bitcoin’s inception, researchers had examined Bitcoin market rigorously from several perspectives.

The first branch focused on assessing the Bitcoin market. Few studies investigated the speculation of Bitcoin [17–20]; Others investigated the efficiency of the Bitcoin market. Through return data in Bitcoin market, Urquhart [21] found empirical evidence to argue an efficient market. However, more research argued the inefficiencies of Bitcoin. For example, Gandal et al. [22] and Sensoy [23] argued the inefficiencies in Bitcoin market and pointed out that suspicious trades might be a source of inefficiencies. Bariviera [24] and Charfeddine et al. [25] discovered long-term dependences in several cryptocurrencies including the Bitcoin. Köchling et al. [26] pointed out that the pricing efficiency of Bitcoin spot prices increased after the introduction of Bitcoin futures. And later, Baur et al., Kapar et al. and Fassas et al. [27–29] provided empirical evidence on the linkages between Bitcoin spot and futures. Dimitrova et al. [30] investigated the efficiency of the Bitcoin market and pointed out the existence of anti-persistent memory in the BTC-USD series. A recent study done by Nikolova et al. [31] further pointed out that the volatility in cryptocurrencies changed faster than in traditional assets, and much faster than in forex pairs.

A second branch investigated the connections between Bitcoin market and other markets. For example, Shahzad et al. and Wang et al. [32, 33] argued that Bitcoin and other cryptocurrencies may act as “safe haven” commodities in global financial markets. Baur et al. [34] analyzed the connections between Bitcoin and traditional assets. The results indicated that Bitcoin was not mainly used as an alternative currency or medium of exchange. Beneki et al. and Walid et al. [35, 36] paid attention to the relationships inside different cryptocurrency markets, i.e., Bitcoin and Ethereum markets. Based on the fact of volatility transmission, the authors suggested possible trading strategies. Other studies had analyzed the determinants of the Bitcoin price from external factors. For example, macroeconomic and monetary factors, as well as factors related to securities. Nevertheless, these results confirmed another phenomenon that price was determined by speculation, supply or demand of Bitcoin [37–40]. A recent study done by Núñez and his colleges [41] made comprehensive investigations on the relationships between the Bitcoin and seven major exchange rates, i.e., RMB, GBP and HKD, etc. Studies also found evidence of micro or macro fundamental factors that affect Bitcoin price. For example, Neves [42] suggested that investment attractiveness had a prominent role in Bitcoin price formation, while other researchers [3, 34, 43, 44] argued the stock market, exchange rate, gold, oil, Economic Policy Uncertainty (EPU), and the Geopolitical Risk Index, etc.

A Third branch might be generalized as Bitcoin market forecasting. Based on the fact the Bitcoin market became a common financial market, researchers also investigated on the issue of whether Bitcoin market was predictable. Despite Urquhart [21] argued the weak form efficiency, numerous studies found empirical evidence on the predictability of Bitcoin return from several aspects, i.e., trading volume, exchange rates, mining rate, revenue, as well as several market indexes [45–54], etc. Based on the time-varying long memory properties in Bitcoin market, Bariviera et al. [55] investigated the Bitcoin volatility forecasting. Recently, García-Medina et al. [56] and García-Medina et al. [57] argued the significant application values of the transfer entropy in the market for cryptocurrencies.

Behavior finance developed rapidly in recent years and showed the importance of individual investor towards financial markets [58, 59]. For example, Han et al., Yao et al., Kou et al. and Li et al. [60–63] proved the importance of investor attention in commodity markets both in linear and non-linear aspects. Chen et al. and Zhang et al. [64, 65] added empirical evidence of investor attention to international financial markets. Connections between investor attention and volatility were also focused by researchers. For example, Audrino et al. [59] pointed the importance of investor attention for volatility and argued that after incorporating investor attention, the accuracy of volatility forecasting significantly improved during out-of-sample period. The academic area also concentrated on the spillover effects from investor attention to financial markets. For example, Wu et al. [66] confirmed the spillovers of investor attention in FX market, and Yin er al. [67] confirmed the effects from oil markets to stock market, etc.

More recently, investor attention was proved to be an important factor in explaining and forecasting return and volatility in digital cryptocurrency markets [3, 68–70]. A bi-directional causal relationship regarding investor attention and Bitcoin return was found by Dastgir et al. [71]. Karalevicius et al. and Garcia et al. [72, 73] suggested that considering search volume surely increased predictive power of Bitcoin in the short term. Twitter was used by several studies to measure investor attention. For example, Shen et al., Philippas et al. and Choi [69, 74, 75] argued that Twitter surely explained the changes of Bitcoin market in several perspectives, i.e., return, volatility and liquidity. Similar with other studies, high frequency data was also concerned. For example, Guégan et al. [76] found statistically significant relationships between investor sentiment and Bitcoin returns. Recently, Ibikunle et al. [70] investigated the relationships between investor attention and price discovery in cryptocurrency markets. In addition to Twitters and Google Trends, researchers had also found alternative variables to measure investor attention. Sabah [77] discovered that the number of new venues could be used to represent the investor attention and argued the importance of the new variable to volatility.

Existing investigations showed that numerous studies have done regarding the Bitcoin market, but studies connected the Bitcoin market with investor attention seemed limited and simple, failing to comprehensively explore the explanatory and predictive power of investor attention. In this paper, we provide more empirical evidence to support the novel variable, i.e., investor attention, and explore the sophisticated relationships between Bitcoin and investor attention. Specifically, we investigated the connections between investor attention and return, as well as volatility of Bitcoin. And in this paper, we make an important step to forecast the Bitcoin market based on investor attention. Moreover, to the best of our knowledge, it is the first attempt to construct economic portfolios in weekly frequency to explore the economic values of incorporating investor attention in Bitcoin market.

## Data

**Fig 1:**
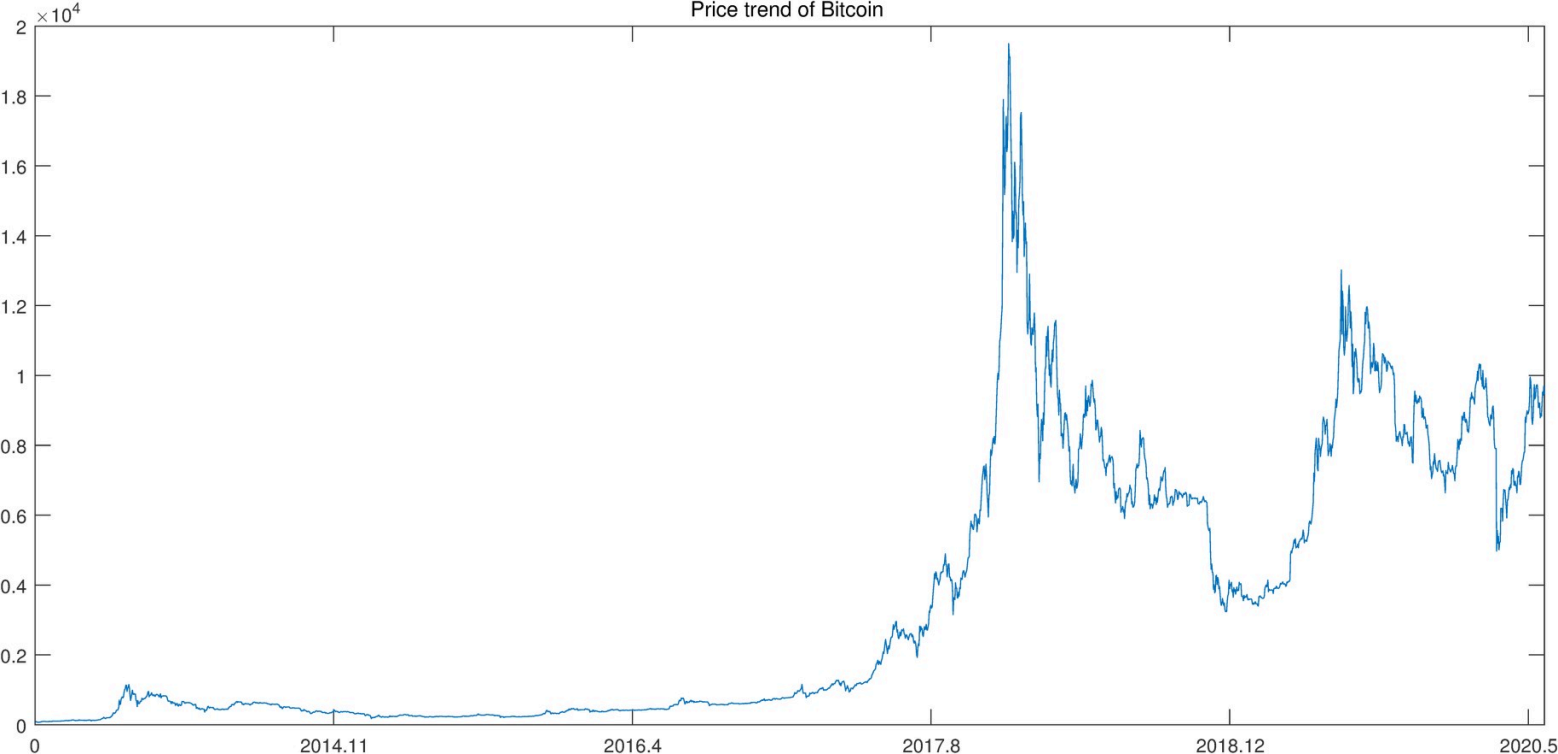
Bitcoin price from July 2013 to May 2020.

Due to data availability, we collect the data of Bitcoin prices from July 1, 2013 to May 31, 2020 in daily frequency. The data of Bitcoin prices are obtained for free from CoinMarketCap (https://coinmarketcap.com/). The price trend of Bitcoin is shown in Fig 1.

As shown in Fig 1, it is clear that from 2013 to 2016, the Bitcoin was at a low price. With the establishment of exchanges for cryptocurrency in 2017, the price of Bitcoin increased sharply. The price showed a downward trend in 2018 and later in the first half of 2019, the price increased. With the outbreak of Covid-19 in 2020, the price increased. Based on these prices, we calculate the average closing prices in one week to represent the weekly Bitcoin price and then transfer these prices to weekly returns. Furthermore, we calculate the weekly realized volatility according to Andersen et al. [78] by daily returns. Some basic descriptive statistics of weekly return and realized volatility are shown in Table 1.

**Table 1:** Descriptive statistics of Bitcoin return and realized volatility.

	Mean	Max	Min	Skewness	Kurtosis	Std. dev
Return	0.0182	0.7166	-0.2907	1.4522	10.4438	0.1024
Realized volatility	0.0130	0.2351	0.0000	5.7271	44.5640	0.0245

As shown in Table 1, the mean value for return is 0.0182 while the mean value for realized volatility is 0.0130. The maximized values of the series are 0.7166 and 0.2351, while the minimized values are -0.2907 and 0.00002, respectively. The standard deviation of realized volatility is 0.0245, while 0.1024 for Bitcoin return. The non-zero skewness and excess kurtosis of the two series indicate that return and realized volatility have characteristics as commonly financial assets [79, 80].

In line with numerous previous studies, we select Google Search Volume Index to represent investor attention [66, 67, 71]. The data for investor attention can be freely achieved from Google Trends (http://www.Google.com/trends). The Google Search Volume Index shows the percentage of search volumes on certain keyword relative to the total number of searches over a given period. The Index shows several advantages to represent the investor attention. First, Internet users commonly use one search engine to collect information, and Google continues to be the most favorite one around the world; Second, and more critically, search can help to avoid the problems resulted from indirect proxies, such as excess return [81], turnover [82], news and headlines [83]; Third, Google search intensity provides a reasonable measure of acquisition of publicly available information from a wide range of sources, providing investors with a highly diversified information set. Therefore, we choose investor attention represented by Google Search Volume Index to conduct follow-up investigations regarding the Bitcoin market.

In this section, we briefly summarize the characteristics of investor attention on Bitcoin. We search the keyword “Bitcoin” in Google Trends. Two types of investor attention on the keyword of “Bitcoin” are downloaded, i.e., monthly data from July 2013 to May 2020 and the daily data for each month during the same period. As mentioned above, Google Search Volume Index is the percentage within a given time period. Therefore, the daily data must be standardized by the weight of the corresponding monthly data. Then, we calculate the average daily search volume index in one week to represent the weekly investor attention, and then calculate the return of these weekly investor attention for further empirical research. Some basic descriptive statistics of investor attention on “Bitcoin” are shown in Table 2.

**Table 2:** Descriptive statistics of investor attention on “Bitcoin”.

	Mean	Max	Min	Skewness	Kurtosis	Std. dev
Investor attention	0.0300	1.5011	-0.5202	2.2058	12.3298	0.2483

Compared with the results in Table 1, it is obvious that difference between the maximized and the minimized value of investor attention, as well as the standard deviation of investor attention are much higher than that of the Bitcoin market. The value of standard deviation to mean is even higher than Bitcoin market. Therefore, it is also high for volatility of investor attention.

**Fig 2:**
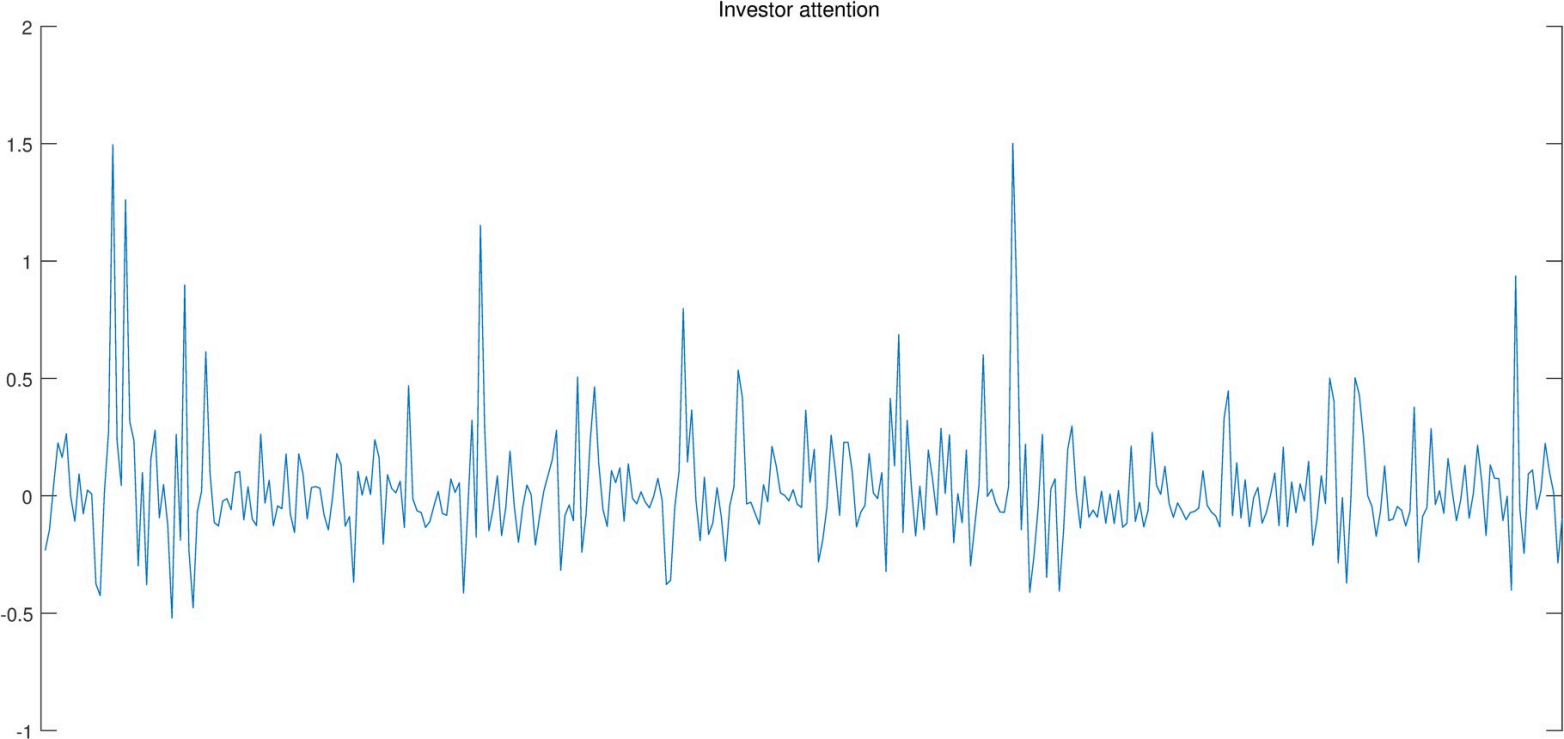
Investor attention.

**Fig 3:**
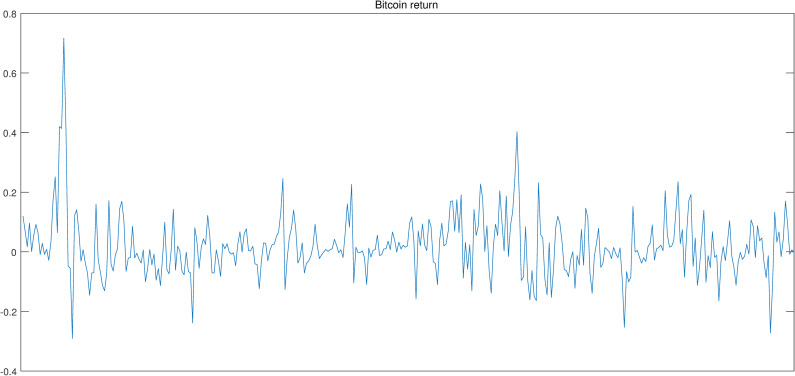
Bitcoin return.

**Fig 4:**
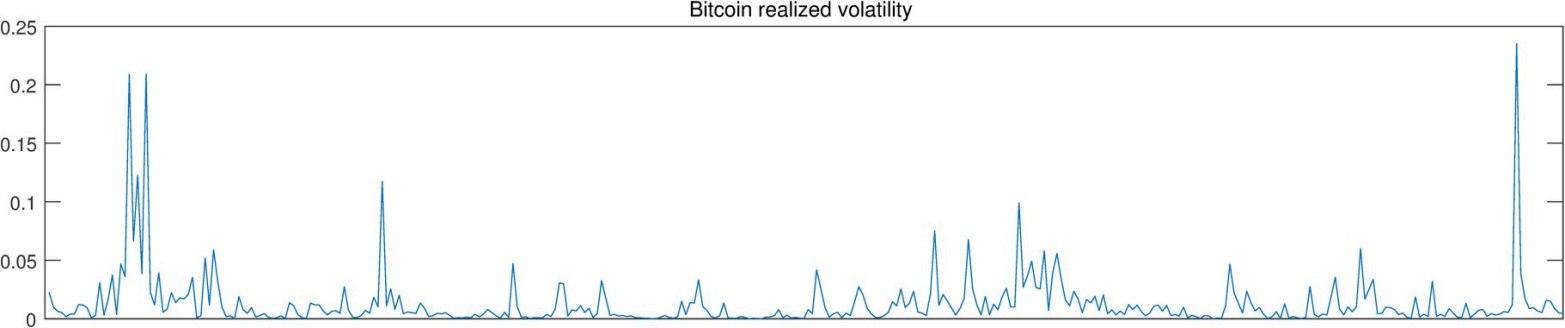
Realized volatility.

Figs 2–4 show the above-mentioned three series, i.e., Bitcoin return, realized volatility and investor attention. Intuitively, investor attention shows same tendency with Bitcoin return and realized volatility. Thus, investor attention may be the granger cause for the other two series.

In the subsequent section, we adopt the VAR model to analyze the correlations between investor attention and Bitcoin market. The prerequisite of VAR model is that the selected series should be stationary. Thus, we implement the ADF stationary test before VAR modelling. The results are shown in the following Table 3.

**Table 3:** ADF test of Bitcoin return, realized volatility and investor attention.

	Type	t-statistic
Return	None	-11.9133***
	Intercept	-12.1432***
	Trent and Intercept	-12.1497***
Realized volatility	None	-4.6227***
	Intercept	-8.6132***
	Trent and Intercept	-8.6409***
Investor attention	None	-17.0632***
	Intercept	-17.2786***
	Trent and Intercept	-17.2794***

In the above Table 3, Intercept, Trend and Intercept, and None represent three types of the Augmented Dickey–Fuller (ADF) test, respectively.

*** represents that the t-statistic is significant at 1%level. The null hypothesis of the ADF test assumes that the series has a unit root.

According to the ADF test results, the null hypothesis for all the three series is rejected. In other words, all the three series are stationary, and thus, can be used for VAR modelling.

## In-sample analyze

### Granger causality

In this section, we conduct the basic Granger causality test to certify whether the linear causality relationships between investor attention and Bitcoin market exist, i.e., return and realized volatility. Standard Granger causality tests in this paper can be summarized by the following Eqs (1)–(4), respectively.

$$R_{t}=\alpha_{01}+\alpha_{11}R_{t-1}+\cdots +\alpha_{n1}R_{t-n}+\beta_{11}Att_{t-1}+\cdots +\beta_{n1}Att_{t-n}+\varepsilon_{t}$$ (1)

$$Att_{t}=\alpha_{02}+\alpha_{12}R_{t-1}+\cdots +\alpha_{n2}R_{t-n}+\beta_{12}Att_{t-1}+\cdots +\beta_{n2}Att_{t-n}+e_{t}$$ (2)

$$V_{t}=\alpha_{03}+\alpha_{13}V_{t-1}+\cdots +\alpha_{n3}V_{t-n}+\beta_{13}Att_{t-1}+\cdots +\beta_{n3}Att_{t-n}+\mu_{t}$$ (3)

$$Att_{t}=\alpha_{04}+\alpha_{14}V_{t-1}+\cdots +\alpha_{n4}V_{t-n}+\beta_{14}Att_{t-1}+\cdots +\beta_{n4}Att_{t-n}+\upsilon_{t}$$ (4)

In the above equations, *R*_*t*_, *V*_*t*_, and *Att*_*t*_ represent the Bitcoin return, realized volatility, and investor attention at week t, respectively. *α*_01_, *α*_02_, *α*_03_, and *α*_04_, represent the constants in the equations, *e*_*t*_, *ε*_*t*_, *μ*_*t*_, and *v*_*t*_ mean the error terms. (*α*_11_,…,*α*_*n*1_, *β*_11_,…*β*_*n*1_), (*α*_12_,…,*α*_*n*2_, *β*_12_,…*β*_*n*2_), (*α*_13_,…,*α*_*n*3_, *β*_13_,…*β*_*n3*_) and (*α*_14_,…,*α*_*n*4_, *β*_14_,…*β*_*n*4_) represent the coefficients for lagged variables in Eqs (1), (2), (3), and (4), respectively. The null hypothesis of granger causality tests for above equations is that the related coefficients are equal to 0. Specifically, we adopt *χ*^2^ statistic to check whether the null hypothesis should be rejected. In other words, we depend on the significance value of *χ*^2^ to determine the granger causality between investor attention and the Bitcoin return, as well as realized volatility. In this paper, we set the lag length to be 4 and choose the time period from August 5, 2013 to June 4, 2017 for in-sample analyze. And thus, the remaining time period from June 4, 2017 to May 31, 2020 is used for out-of-sample prediction. The results of granger causality tests for in-sample period are shown in the following Table 4.

**Table 4:** Granger causality test results between investor attention and Bitcoin.

Equation	Excluded	*χ*^2^-statistic
Return	Investor attention	10.239**
Investor attention	Return	19.829***
Realized volatility	Investor attention	38.03***
Investor attention	Realized volatility	4.310

Note

*, **, *** denote significance at 10%, 5% and 1% level, respectively.

As shown in Table 4, the results reveal important facts in Bitcoin market. There exists a bi-directional granger causality between Bitcoin return and investor attention, while a unidirectional granger causality from investor attention to realized volatility is certified. To sum up, granger causality test results identify a fact that investor attention is a non-negligible factor in the Bitcoin market.

### VAR analysis

The VAR model is a linear predictive model, which allows the variables to be forecasted by past values. According to previous studies [84–86], the VAR model is widely used in estimating and predicting financial assets. For the perfect fitness of the VAR model on assets, we adopt the VAR model to basically investigate the relationships between investor attention and Bitcoin market, i.e., return and realized volatility. The VAR model can be generalized by the following Eq (5) according to Han et al. [87],

$$X_{t}=c+\sum_{i=1}^{p}\beta_{i}X_{t-i}+\varepsilon_{t}$$ (5)

where *X*_*t*_ contains two parts, the first part refers to the Bitcoin market, i.e., return or realized volatility, while the second part refers to the investor attention. p represents the lag length inside a certain VAR model and *β*_*i*_ is the coefficient for the lagged term *X*_*t−i*_. The VAR specification helps to understand the reactions of Bitcoin return or realized volatility to the shock from investor attention under the framework of impulse response function, and vice versa. In the construction of the VAR model, an important step is to quantify the lag length in the model. We report the lag length selection process for the VAR model in the following Table 5.

**Table 5:** Lag length selection for VAR model.

Lag	LR	FPE	AIC	SC
Panel A: VAR lag length selection for Bitcoin return				
1	87.5049	0.000487	-1.9505	-1.8846*
2	7.7458	0.000488	-1.9501	-1.8403
3	12.3890	0.000481	-1.9632	-1.8096
4	8.7808	0.000480*	-1.9661*	-1.7685
5	5.7436	0.000483	-1.9602	-1.7187
6	3.6836	0.000489	-1.9484	-1.6630
7	11.2247*	0.000483	-1.9589	-1.6297
Panel B: VAR lag length selection for Bitcoin realized volatility				
1	45.2735	3.08e-05	-4.7137	-4.6478
2	33.5268	2.86e-05	-4.7876	-4.6778*
3	12.8820	2.82e-05	-4.8022	-4.6485
4	28.5292*	2.65e-05*	-4.8626*	-4.6650
5	1.6454	2.70e-05	-4.8447	-4.6032
6	1.6708	2.75e-05	-4.8269	-4.5415
7	7.8523	2.75e-05	-4.8275	-4.4982

Note: LR is sequential modified LR test statistic, FPE represent the final prediction error, AIC refer to the Akaike information criterion, SC means Schwarz information criterion.

* indicates lag order selected by the criterion.

Thus, in this paper, according to FPE and AIC criteria, we set the lag length to 4, namely, p is equal to 4 in Eq (5) for both Bitcoin return and realized volatility. We conduct the VAR analyze for the in-sample period for return and realized volatility, respectively. And the results are shown in Table 6.

**Table 6:** VAR regression results of Bitcoin market and investor attention.

Panel A: Bitcoin return			Panel B: Bitcoin realized volatility		
	R_t_	Att_t_		V_t_	Att_t_
R_t-1_	0.4506***	0.6882***	V_t-1_	0.0318	-0.2631
	(0.0727)	(0.1982)		(0.0700)	(0.8814)
R_t-2_	0.0177	0.2770	V_t-2_	0.2969***	1.1833
	(0.0785)	(0.2140)		(0.0677)	(0.8520)
R_t-3_	-0.1316*	-0.0977	V_t-3_	-0.0462	-0.7950
	(0.0786)	(0.2140)		(0.0674)	(0.8486)
R_t-4_	0.0811	0.1193	V_t-4_	0.2455***	0.6316
	(0.0712)	(0.1940)		(0.0662)	(0.8329)
Att_t-1_	0.0653**	0.0045	Att_t-1_	0.0205***	0.1366*
	(0.0264)	(0.0719)		(0.0058)	(0.0732)
Att_t-2_	-0.0108	-0.1039	Att_t-2_	0.0052	-0.0459
	(0.0264)	(0.0718)		(0.0059)	(0.0740)
Att_t-3_	0.0382	-0.0982	Att_t-3_	0.0261***	-0.0388
	(0.0262)	(0.0713)		(0.0059)	(0.0738)
Att_t-4_	-0.0339	-0.1802**	Att_t-4_	0.0125**	-0.1498**
	(0.0262)	(0.0713)		(0.0059)	(0.0746)
Intercept	0.0096	0.0233**	Intercept	0.0036**	0.0246
	(0.0065)	(0.0176)		(0.0017)	(0.0212)
R^2^	0.2753	0.1239	R^2^	0.4042	0.0574

Note: This table reports the results of VAR analysis between Bitcoin market and investor attention. The VAR estimation results for Bitcoin return and realized volatility are reported by Panel A and Panel B, respectively. Panel A is used to present the results of VAR estimations of Bitcoin return and investor attention, while Panel B is used to present the results of VAR estimations of Bitcoin realized volatility and investor attention. The value in the bracket represents the standard error.

*, **, and *** denote significance at 10%, 5% and 1% level, respectively.

In the above Table 6, several interesting discoveries can be found. Firstly, investor attention in the last period had significant positive impacts on current Bitcoin market both in return and realized volatility. However, such influence disappeared quickly for Bitcoin return, as other lags, i.e., 2, 3, 4, are insignificant. However, investor attention may affect the Bitcoin realized volatility for several period, as the coefficients of investor attention at the third and four lags are still significant. Second, current investor attention is also positively affected by Bitcoin returns in the last week, as R_t-1_ is significant for the equation of attention. However, such influence quickly disappears as the coefficients for other lags are insignificant. In contrast, realized volatility does not show any affects to investor attention regarding our VAR model, as all the coefficients do not significant at 10% level.

**Fig 5:**
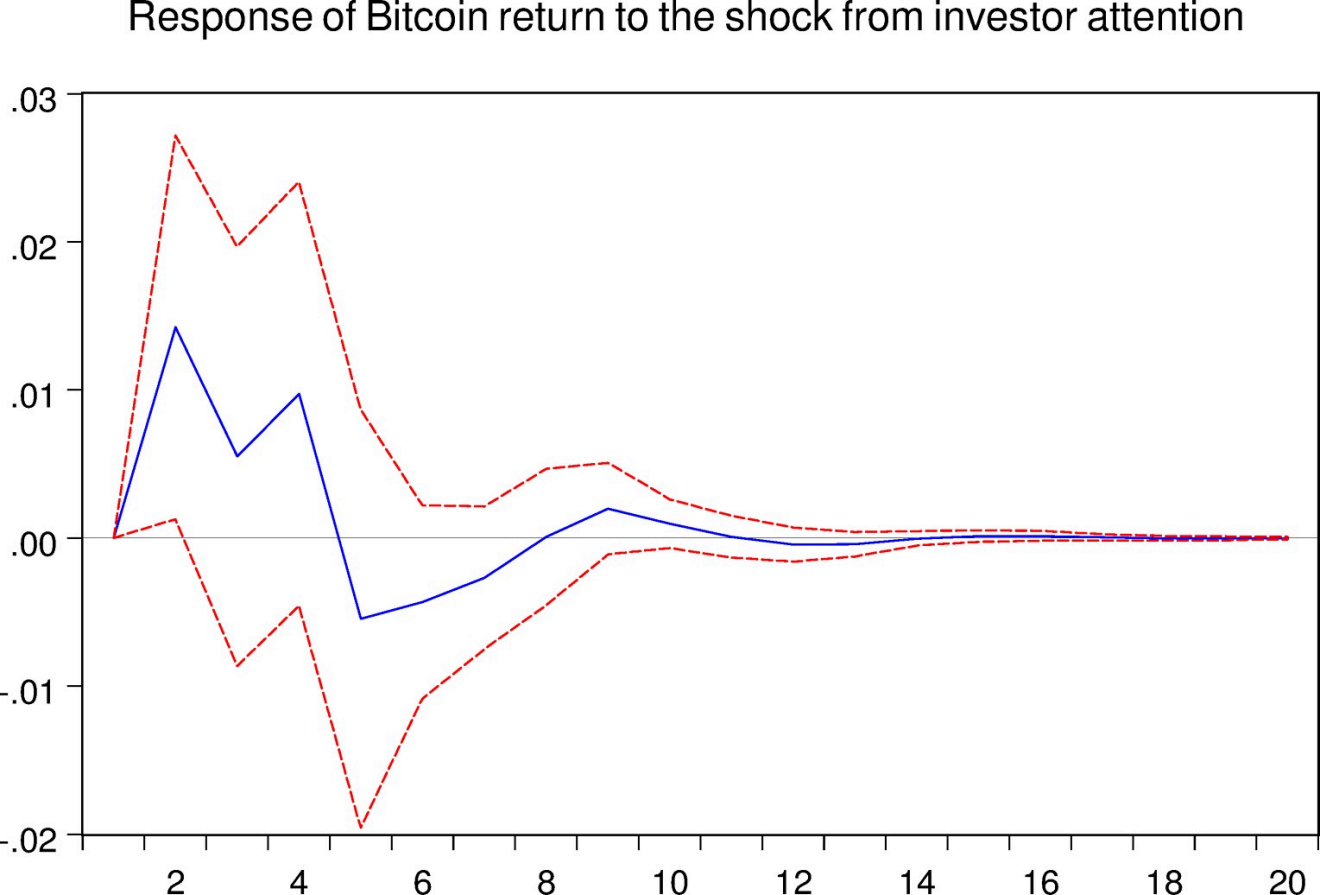
Response of Bitcoin return to the shock from investor attention. The blue solid-line is the impulse response to Cholesky one standard deviation innovations, while the red dotted-line is ninety five percent confidence interval for highest probability density. The X-axis represents the duration of shock, while the Y-axis represents the magnitude of such shock.

**Fig 6:**
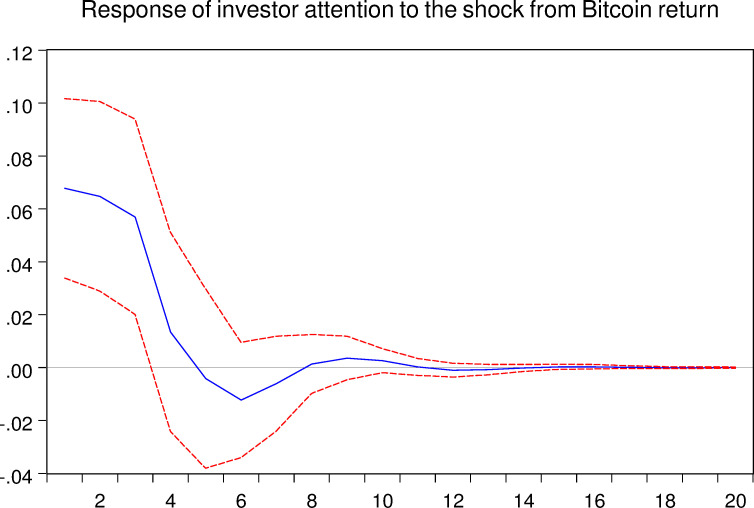
Response of investor attention to the shock from Bitcoin return. The blue solid-line is the impulse response to Cholesky one standard deviation innovations, while the red dotted-line is ninety five percent confidence interval for highest probability density. The X-axis represents the duration of shock, while the Y-axis represents the magnitude of such shock.

**Fig 7:**
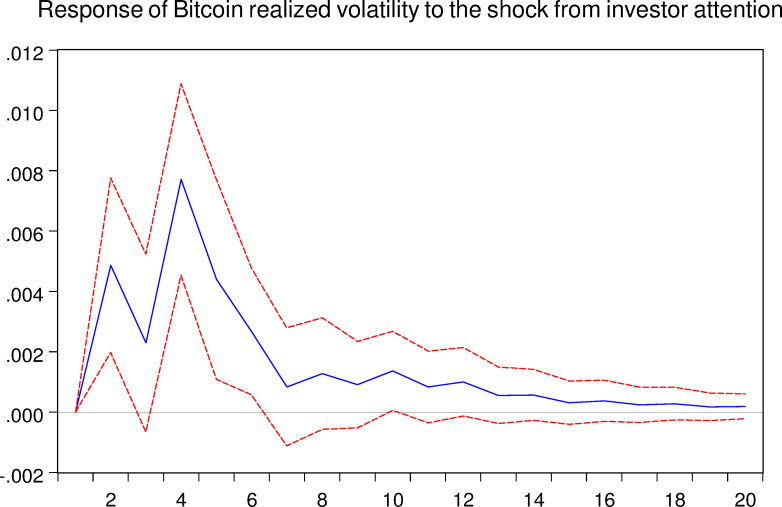
Response of Bitcoin realized volatility to the shock from investor attention. The blue solid-line is the impulse response to Cholesky one standard deviation innovations, while the red dotted-line is ninety five percent confidence interval for highest probability density. The X-axis represents the duration of shock, while the Y-axis represents the magnitude of such shock.

**Fig 8:**
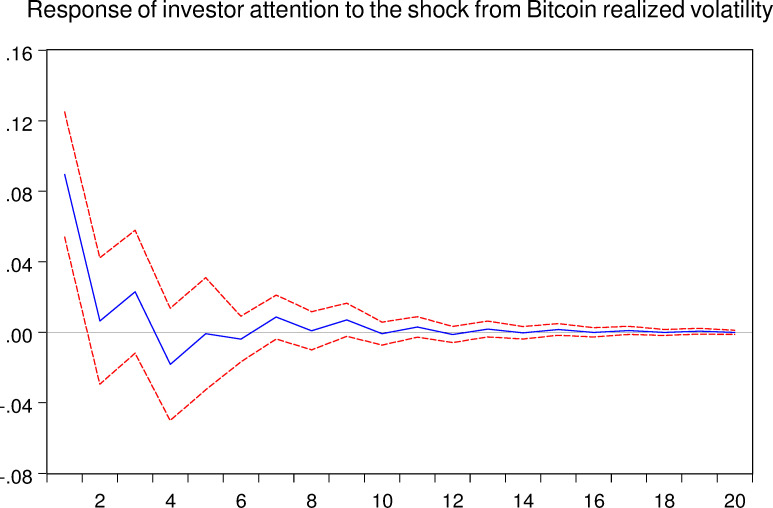
Response of investor attention to the shock from Bitcoin realized volatility. The blue solid-line is the impulse response to Cholesky one standard deviation innovations, while the red dotted-line is ninety five percent confidence interval for highest probability density. The X-axis represents the duration of shock, while the Y-axis represents the magnitude of such shock.

As mentioned above, the VAR model allows us to analyze the response of one certain variable to the shock from another variable. Thus, in this part, we implement the impulse response analyze between Bitcoin asset and investor attention. And the related results are shown in the following Figs 5–8, respectively.

As shown in Figs 5 and 7, we can conclude that the investor attention indeed affects the Bitcoin market both in return and realized volatility, respectively. For the Bitcoin return, shock from investor attention last for approximately 12 weeks. For the Bitcoin realized volatility, shock may last 16 weeks. Therefore, the effects of investor attention in Bitcoin market are worth for further discussions.

### High order moment

Inspired by other researchers exploring the relationships between high order moments of investor attention and assets [88], we further conduct the related empirical investigations on this issue. Specifically, we consider the squared and cubic terms of investor attention following Han’s empirical process exploring the non-linear relationships between FX market and investor attention [88]. The preliminary results show that the cubic terms of investor attention can better explain the changes in Bitcoin market, while the squared terms cannot. Thus, in this section, only the results for cubic terms are reported. The model specifications for return and realized volatility are shown as follows in Eqs (6) and (7) according to Han et al. [88].

$$R_{t}=\alpha_{01}+\sum_{i=1}^{4}\alpha_{i1}R_{t-i}+\sum_{i=1}^{4}\beta_{i1}Att_{t-i}+\sum_{i=1}^{4}\gamma_{i1}Att_{t-i}^{3}+\varepsilon_{t}$$ (6)

$$V_{t}=\alpha_{02}+\sum_{i=1}^{4}\alpha_{i2}V_{t-i}+\sum_{i=1}^{4}\beta_{i2}Att_{t-i}+\sum_{i=1}^{4}\gamma_{i2}Att_{t-i}^{3}+e_{t}$$ (7)

In the above specifications, the γ_ij_ reflects the influence of high order moment *Att*_*t−i*_ of investor attention to the current Bitcoin return or volatility. (β+γ) measure the overall impacts of lagged attention on current Bitcoin return or volatility. The estimation results of the two models are shown in Table 7.

**Table 7:** The estimation results of higher moment.

Panel A: Bitcoin return		Panel B: Bitcoin realized volatility	
	R_t_		V_t_
R_t-1_	0.3566***	V_t-1_	-0.0743
	(0.0733)		(0.0670)
R_t-2_	-0.0862	V_t-2_	0.3110***
	(0.0733)		(0.0628)
R_t-3_	-0.1514**	V_t-3_	-0.0391
	(0.0734)		(0.0626)
R_t-4_	0.0372	V_t-4_	0.1675***
	(0.0660)		(0.0616)
Att_t-1_	-0.0367	Att_t-1_	0.0202***
	(0.0329)		(0.0075)
Att_t-2_	-0.0432	Att_t-2_	-0.0026
	(0.0325)		(0.0075)
Att_t-3_	-0.0901***	Att_t-3_	-0.0068
	(0.0325)		(0.0075)
Att_t-4_	-0.0154	Att_t-4_	-0.0065
	(0.0341)		(0.0075)
Att^3^_t-1_	0.1494***	Att^3^_t-1_	-0.0067
	(0.0285)		(0.0065)
Att^3^_t-2_	0.0756**	Att^3^_t-2_	0.0115*
	(0.0298)		(0.0062)
Att^3^_t-3_	0.1693***	Att^3^_t-3_	0.0403***
	(0.0293)		(0.0062)
Att^3^_t-4_	-0.0113	Att^3^_t-4_	0.0310***
	(0.0329)		(0.0071)
Intercept	0.0060	Intercept	0.0041***
	(0.0059)		(0.0015)
R^2^	0.4094	R^2^	0.5130

Note: There are two parts in this table, which report the results of investor attention on Bitcoin return and realized volatility, respectively. The table includes the estimation results for lagged Bitcoin return (*R*_*t−i*_, *i* = 1,2,3,4), lagged Bitcoin realized volatility (*V*_*t−i*_, *i* = 1,2,3,4), lagged investor attention (*Attt*_*t−i*_, *i* = 1,2,3,4) and lagged cubic term of investor attention (${Att}_{t-i}^{3},i=1,2,3,4$). The value in the bracket represents the standard error.

*, **, and *** denote significance at 10%, 5% and 1% level, respectively.

As shown in Table 7, three facts may be identified. First, considering the high order moment of investor attention, linear term represented by Att_t-i_ (i = 1,2,3,4) of investor attention still has significant impacts on Bitcoin return and volatility, which supports the results obtained from VAR linear analysis. Second, the total impacts of investor attention on Bitcoin return may be quantified. The linear terms and the cubic terms of investor attention are significant at a same lag length of 3. Besides, we may also notice that the absolute value of coefficients of cubic investor attention are greater than that of the linear terms, which may reflect a simple reality that a change in investor attention may has a positive impact on the Bitcoin return in three weeks later. Third, cubic terms of investor attention indeed have explanatory powers on the current Bitcoin realized volatility, as the cubic terms of investor attention in the 2, 3 and 4 lags all are significant. However, the total impacts of investor attention on Bitcoin realized volatility are not clear as the linear terms and the cubic terms do not significant at a same lag.

### Asymmetry

Vozlyublennaia [89] and Andrei et al. [90] pointed out that past declines or negative changes in asset performance are considered as "bad news" and may cause great attention to the asset. Based on this insight, we consider the characteristics of the information received by investors in the Bitcoin market, measured by the signs of past changes in Bitcoin earnings, to further quantify the asymmetric relationship between investor attention and Bitcoin. The model specification for analyzing asymmetric relationships between investor attention and Bitcoin return can be generalized by the following Eq (8) according to Han et al. [87] and Han [88] et al.

$$R_{t}=\alpha_{01}+\sum_{i=1}^{4}\alpha_{i1}R_{t-i}+\sum_{i=1}^{4}\beta_{i1}Att_{t-i}+\sum_{i=1}^{4}\eta_{i1}Att_{t-i}*D(R_{t-i}<0)+\varepsilon_{t}$$ (8)

In the above specification, *η*_*i*_ measures the magnitude of asymmetrical effect, and (*β*+*η*) measure the total impacts of investor attention. The estimation results are shown in Table 8.

**Table 8:** Asymmetrical effects of investor attention on Bitcoin return.

Bitcoin return	
Variables	Coefficient
R_t-1_	0.4395***
	(0.0616)
R_t-2_	0.0190
	(0.0819)
R_t-3_	-0.1519*
	(0.0820)
R_t-4_	0.0374
	(0.0762)
Att_t-1_	0.0604*
	(0.0326)
Att_t-2_	0.0056
	(0.0324)
Att_t-3_	0.0659**
	(0.0321)
Att_t-4_	-0.0195
	(0.0316)
Att_t-1_*D(R_t-1_)	-0.0114
	(0.0616)
Att_t-2_*D(R_t-2_)	-0.0690
	(0.0611)
Att_t-3_*D(R_t-3_)	-0.1071*
	(0.0610)
Att_t-4_*D(R_t-4_)	-0.0591
	(0.0608)
Intercept	0.0077
	(0.0067)
R^2^	0.2474

Note: This table reports the asymmetrical effects between investor attention and Bitcoin return. Volatility is not reported as all the realized volatilities are positive. The table contains the estimation results for lagged Bitcoin return (*R*_*t−i*_, *i* = 1,2,3,4), lagged investor attention (*Att*_*t−i*_, *i* = 1,2,3,4) and lagged asymmetrical term (*Att*_t−i_*D(*R*_*t−i*_), *i* = 1,2,3,4). The value in the bracket represents the standard error.

*, **, and *** denote significance at 10%, 5% and 1% level, respectively.

As shown in Table 8, an interesting discovery is found, attention and the asymmetrical term are both negatively significant in the lag length of 3. Thus, asymmetric effect between investor attention and Bitcoin return surely exists and can be quantified. A negative return surely affects the investor attention and ultimately affects the Bitcoin after 3 weeks.

### Interactive relationship

Inspired by existing research [87], we have also incorporated the interactive terms of lagged investor attention with Bitcoin return or realized volatility into our models to further explore the relationships between investor attention and Bitcoin market. The related model specifications for this issue are shown as follows in (9) and (10) according to Han [87] and Han [88],

$$R_{t}=\alpha_{01}+\sum_{i=1}^{4}\alpha_{i1}R_{t-i}+\sum_{i=1}^{4}\beta_{i1}Att_{t-i}+\sum_{i=1}^{4}\lambda_{i1}Att_{t-i}*R_{t-i}+\varepsilon_{t}$$ (9)

$$V_{t}=\alpha_{02}+\sum_{i=1}^{4}\alpha_{i2}V_{t-i}+\sum_{i=1}^{4}\beta_{i2}Att_{t-i}+\sum_{i=1}^{4}\lambda_{i2}Att_{t-i}*V_{t-i}+e_{t}$$ (10)

where coefficients λ_ij_ for the interactive term in Eqs (9) and (10) measures the impact of interactive term on future Bitcoin market, i.e., return and realized volatility. The results of the two models are shown in Table 9.

**Table 9:** The results of interactive effects.

Panel A: Bitcoin return		Panel B: Bitcoin realized volatility	
R_t-1_	0.4294***	V_t-1_	0.1048
	(0.0844)		(0.0778)
R_t-2_	0.0923	V_t-2_	0.0891
	(0.0908)		(0.0782)
R_t-3_	-0.0684	V_t-3_	0.0853
	(0.0922)		(0.0732)
R_t-4_	0.2453***	V_t-4_	-0.0356
	(0.0882)		(0.0723)
Att_t-1_	0.0662**	Att_t-1_	0.0174***
	(0.0662)		(0.0058)
Att_t-2_	0.0168	Att_t-2_	-0.0015
	(0.0168)		(0.0057)
Att_t-3_	0.0399	Att_t-3_	0.0291***
	(0.0281)		(0.0057)
Att_t-4_	-0.0164	Att_t-4_	-0.0093
	(0.0309)		(0.0063)
R_t-1_*Att_t-1_	0.0094	V_t-1_*Att_t-1_	0.0567
	(0.0094)		(0.1039)
R_t-2_*Att_t-2_	-0.3246**	V_t-2_*Att_t-2_	0.3081***
	(0.1326)		(0.0960)
R_t-3_*Att_t-3_	-0.1485	V_t-3_*Att_t-3_	-0.2264**
	(0.1287)		(0.0946)
R_t-4_*Att_t-4_	-0.3144**	V_t-4_*Att_t-4_	0.7330***
	(0.1255)		(0.0957)
Intercept	0.0096	Intercept	0.0063***
	(0.0065)		(0.0016)
R^2^	0.2734	R^2^	0.5388

Note: There are two parts in this table, which respectively report the results of model (9) and (10). The table includes the estimation results of lagged Bitcoin return (*R*_*t−i*_, *i* = 1,2,3,4), lagged Bitcoin realized volatility (*V*_*t−i*_, *i* = 1,2,3,4), lagged investor attention (*Att*_*t−i*_, *i* = 1,2,3,4) and lagged interactive term of investor attention with Bitcoin market. The value in the bracket represents the standard error.

*, **, and *** denote significance at 10%, 5% and 1% level, respectively.

As shown in Table 9, if the interactive terms of investor attention and Bitcoin realized volatility are presented in the model specifications, the influence of investor attention on Bitcoin realized volatility can indeed be quantified as investor attention and the interactive terms are significant in the same lag length of 3. However, it can only be conducted that investor attention is an important factor for Bitcoin return. The influence of investor attention could not be quantified, as investor attention and the interact terms do not significant at a same lag length.

### Joint impacts of with other assets

As indicated by existing studies that stock market and the macroeconomic uncertainty are closely related to the Bitcoin market [3, 43, 91]. Therefore, in this section, we control the above-mentioned factors to further explore the relationships of investor attention on Bitcoin market, i.e., return and realized volatility. Specifically, the return for Dow Jones Industrial Index (Dow) and U.S. Equity Market Volatility (Emu) are selected as control variables. The concrete model specifications are as follows in (11) and (12) according to Han et al. [87],

$$R_{t}=\alpha_{01}+\sum_{i=1}^{4}\alpha_{i1}R_{t-i}+\sum_{i=1}^{4}\beta_{i1}Att_{t-i}+\sum_{i=1}^{4}\theta_{i1}Control_{t-i}+\sum_{i=1}^{4}\delta_{i1}Att_{t-i}*Control_{t-i}+\varepsilon_{t}$$ (11)

$$V_{t}=\alpha_{02}+\sum_{i=1}^{4}\alpha_{i2}V_{t-i}+\sum_{i=1}^{4}\beta_{i2}Att_{t-i}+\sum_{i=1}^{4}\theta_{i2}Control_{t-i}+\sum_{i=1}^{4}\delta_{i2}Att_{t-i}*Control_{t-i}+e_{t}$$ (12)

where the *Control*_*t−i*_ represents Dow Jones Industrial Index and U.S. Equity Market Volatility, respectively. The coefficient θ_ij_ represents the effect of control variable on Bitcoin market. The coefficient δ_ij_ estimates the interactive term between investor attention and related assets. The coefficients (θ +δ) reflect the total effects of investor attention on Bitcoin return or realized volatility under the condition of control variables. The regression results are shown in the following Table 10.

**Table 10:** The results for joint impacts.

Panel A: Bitcoin return			Panel B: Bitcoin realized volatility		
	Control (Dow)	Control (Emu)		Control (Dow)	Control (Emu)
R_t-1_	0.4142***	0.4174***	V_t-1_	-0.0248	0.0017
	(0.0764)	(0.0759)		(0.0721)	(0.0724)
R_t-2_	-0.0232	0.0258	V_t-2_	0.2743***	0.2689***
	(0.0820)	(0.0808)		(0.0672)	(0.0680)
R_t-3_	-0.1362*	-0.1027	V_t-3_	-0.0440	-0.0159
	(0.0821)	(0.0822)		(0.0666)	(0.0680)
R_t-4_	0.0577	0.0656	V_t-4_	0.2406***	0.2692***
	(0.0736)	(0.0732)		(0.0659)	(0.0672)
Att_t-1_	0.0699**	0.0637**	Att_t-1_	0.0165***	0.0202***
	(0.0280)	(0.0272)		(0.0060)	(0.0060)
Att_t-2_	0.0052	-0.0035	Att_t-2_	0.0035	0.0070
	(0.0275)	(0.0278)		(0.0059)	(0.0061)
Att_t-3_	0.0472*	0.0507*	Att_t-3_	0.0256***	0.0311***
	(0.0273)	(0.0275)		(0.0058)	(0.0061)
Att_t-4_	-0.0270	-0.0387	Att_t-4_	0.0144**	0.0126**
	(0.0276)	(0.0279)		(0.0059)	(0.0062)
Control_t-1_	1.0328**	0.0009	Control_t-1_	-0.0252	-0.0011
	(0.5106)	(0.0060)		(0.1092)	(0.0013)
Control_t-2_	0.1914	-0.0078	Control_t-2_	-0.0792	-0.0028**
	(0.5332)	(0.0061)		(0.1137)	(0.0013)
Control_t-3_	-0.0724	-0.0010	Control_t-3_	0.1961*	-0.0019
	(0.5376)	(0.0061)		(0.1163)	(0.0013)
Control_t-4_	0.2552	-0.0017	Control_t-4_	0.0195	-0.0012
	(0.5181)	(0.0058)		(0.1125)	(0.0013)
Att_t-1_*Control_t-1_	3.8124*	-0.0403	Att_t-1_*Control_t-1_	0.3492	-0.0166*
	(2.1257)	(0.0420)		(0.4595)	(0.0091)
Att_t-2_*Control_t-2_	0.7102	-0.0814*	Att_t-2_*Control_t-2_	0.5275	-0.0080
	(2.1255)	(0.0419)		(0.4541)	(0.0092)
Att_t-3_*Control_t-3_	2.2097	-0.1114***	Att_t-3_*Control_t-3_	1.3988***	-0.0331***
	(2.1019)	(0.0418)		(0.4486)	(0.0092)
Att_t-4_*Control_t-4_	-0.5975	-0.0557	Att_t-4_*Control_t-4_	1.1206**	-0.0084
	(2.0871)	(0.0420)		(0.4562)	(0.0094)
Intercept	0.0076	0.0116	Intercept	0.0045***	0.0053***
	(0.0067)	(0.0076)		(0.0017)	(0.0019)
R^2^	0.2566	0.2667	R^2^	0.4389	0.4182

Note: This table reports the estimation results for model (11) and (12). The table contains the estimation results of lagged Bitcoin return (*R*_*t−i*_, *i* = 1,2,3,4), lagged investor attention (*Att*_*t−i*_, *i* = 1,2,3,4), lagged control variable (*Control*_*t−i*_, *i* = 1,2,3,4), and the interactive term between lagged investor attention and control variable (*Att*_t−i_*Control_*t−i*_, *i* = 1,2,3,4). The value in the bracket represents the standard error.

*, **, *** denote significance at 10%, 5% and 1% level, respectively.

As shown in Table 10, the interactive terms between investor attention and control variables generate significantly effects on Bitcoin asset. Specifically, for the Bitcoin return, the interactive terms and the investor attention are both significantly positive at a same lag of 1 or 3, which reflects that after controlling the related assets, the investor attention is still an important factor for Bitcoin return and the influence of investor can be quantified. As for the Bitcoin realized volatility, the interactive terms and the investor attention are both significantly positive at a same lag of 1, 3 or 4, the results also indicate that the impacts of investor attention on Bitcoin realized volatility can be quantified.

To sum up, investor attention is the granger cause of changes in Bitcoin market, i.e., return and realized volatility. Besides, it also generates linear and non-linear effects, i.e., asymmetric, interact, higher moment as well as joint impacts with related assets. However, the significant explanatory power does not mean that investor attention could forecast the Bitcoin market in the out-of-sample period. Thus, in the subsequent section, we explore the predictive power of investor attention in Bitcoin market, regarding both return and realized volatility.

## Out-of-sample forecast

As Welch et al. [92] argued that in-sample estimations maybe overfitted. Therefore, one significant variable during in-sample period does not represent a well forecasting power in out-of-sample period. In order to avoid such issue and further explore the relationships between investor attention and Bitcoin market, we conduct several out-of-sample forecasts regarding the Bitcoin return and realized volatility. In this paper, we use a rolling window method to predict Bitcoin return or realized volatility at one certain week based on the data in previous weeks, which means that the rolling window method should drop the most distant data to do parameter estimation after adding a new observation [93]. Hence, the window size keeps fixed when the estimation window rolls forward. We can then obtain the Bitcoin return or realized volatility in the future based on these forecast methods by employing the following Eqs (13) to (21) according to Han et al. [87] and Wang et al. [94],

$$\hat{R_{t+h}}=\hat{\alpha_{01}}+\sum_{i=1}^{4}\hat{\alpha_{i1}}R_{t+h-i}+\sum_{i=1}^{4}\hat{\beta_{i1}}{Att}_{t+h-i}$$ (13)

$$\hat{V_{t+h}}=\hat{\alpha_{02}}+\sum_{i=1}^{4}\hat{\alpha_{i2}}R_{t+h-i}+\sum_{i=1}^{4}\hat{\beta_{i2}}{Att}_{t+h-i}$$ (14)

$$\hat{R_{t+h}}=\hat{\alpha_{01}}+\sum_{i=1}^{4}\hat{\alpha_{i1}}R_{t+h-i}+\sum_{i=1}^{4}\hat{\beta_{i1}}{Att}_{t+h-i}+\sum_{i=1}^{4}\hat{\gamma_{i1}}{Att}_{t+h-i}^{3}$$ (15)

$$\hat{V_{t+h}}=\hat{\alpha_{02}}+\sum_{i=1}^{4}\hat{\alpha_{i2}}V_{t+h-i}+\sum_{i=1}^{4}\hat{\beta_{i2}}{Att}_{t+h-i}+\sum_{i=1}^{4}\hat{\gamma_{i2}}{Att}_{t+h-i}^{3}$$ (16)

$$\hat{R_{t+h}}=\hat{\alpha_{01}}+\sum_{i=1}^{4}\hat{\alpha_{i1}}R_{t+h-i}+\sum_{i=1}^{4}\hat{\beta_{i1}}{Att}_{t+h-i}+\sum_{i=1}^{4}\hat{\eta_{i1}}{Asymmetrical}_{t+h-i}$$ (17)

$$\hat{R_{t+h}}=\hat{\alpha_{01}}+\sum_{i=1}^{4}\hat{\alpha_{i1}}R_{t+h-i}+\sum_{i=1}^{4}\hat{\beta_{i1}}{Att}_{t+h-i}+\sum_{i=1}^{4}\hat{\lambda_{i1}}{Att*R}_{t+h-i}$$ (18)

$$\hat{V_{t+h}}=\hat{\alpha_{02}}+\sum_{i=1}^{4}\hat{\alpha_{i2}}V_{t+h-i}+\sum_{i=1}^{4}\hat{\beta_{i2}}{Att}_{t+h-i}+\sum_{i=1}^{4}\hat{\lambda_{i2}}{Att*V}_{t+h-i}$$ (19)

$$\hat{R_{t+h}}=\hat{\alpha_{01}}+\sum_{i=1}^{4}\hat{\alpha_{i1}}R_{t+h-i}+\sum_{i=1}^{4}\hat{\beta_{i1}}{Att}_{t+h-i}+\sum_{i=1}^{4}\hat{\theta_{i1}}{Control}_{t+h-i}+\sum_{i=1}^{4}\hat{\delta_{i1}}{Att*Control}_{t+h-i}$$ (20)

$$\hat{V_{t+h}}=\hat{\alpha_{02}}+\sum_{i=1}^{4}\hat{\alpha_{i2}}V_{t+h-i}+\sum_{i=1}^{4}\hat{\beta_{i2}}{Att}_{t+h-i}+\sum_{i=1}^{4}\hat{\theta_{i2}}{Control}_{t+h-i}+\sum_{i=1}^{4}\hat{\delta_{i2}}{Att*Control}_{t+h-i}$$ (21)

where h is forecast horizon, representing the week investors would like to forecast. Specifically, the horizon is set as 1 if investors would like to forecast the return or realized volatility in next week, and the horizon is set to 2 if investors would like to estimate the values of the week after next week. All the parameters are estimated by the OLS regression based on in-sample series with fixed rolling window and are updated in each prediction. The nine forecasting equations can be divided into two parts. Eqs (13), (15), (17), (18) and (20) are for Bitcoin return forecasting, while Eqs (14), (16), (19) and (21) are for the realized volatility of Bitcoin, respectively. In this paper, historical average is selected as the benchmark forecasting model. Moreover, Out-of-sample R squared ($R_{oos}^{2}$), mean squared forecast error (MSFE), MSFE-adjusted statistic and the related P value are adopted as criterions. Detailed information of these statistics can be generalized as follows.

The $R_{oos}^{2}$ indicates the proportion reduction in MSFE for using the predictive model compared with the benchmark model. A positive $R_{oos}^{2}$ indicates that the forecast performance of the prediction model outperforms the benchmark model. The $R_{oos}^{2}$ is obtained from the following Eq (22),

$$R^{2}=R_{oos}^{2}=1-\frac{\sum_{k=t+horizon+1}^{T}{\left(R_{k}-\hat{R_{k}}\right)}^{2}}{\sum_{k=t+horizon+1}^{T}{\left(R_{k}-\overset{-}{R_{k}}\right)}^{2}}$$ (22)

where T is the number for out-of-sample. *R*_*k*_ represents the real value, $\hat{R_{k}}$ is the forecasted value by the above-mentioned equations. $\bar{R_{k}}$ is the predicted value of benchmark model. As indicated by previous studies, it is difficult for an individual model to significantly outperform a historical average benchmark model in out-of-sample period. Thus, in this paper, we set historical average model as our benchmark model. The benchmark model is given by Eq (23),

$$\bar{R_{k+1}}=\frac{1}{k}\sum_{s=1}^{k}R_{s}$$ (23)

where $\bar{R_{k+1}}$ represents the forecast value while *R*_*s*_ means the true value in the past. A positive R squared (R^2^) indicates that the predictive model based on investor attention outperforms the benchmark model, i.e., historical average.

The MSFE statistic value is obtained by the equation following (24).

$$MSFE=\frac{\sum_{k=t+horizon+1}^{T}{\left(R_{k}-\hat{R_{k}}\right)}^{2}}{T-t-horizon}$$ (24)

We also consider a decomposition of MSFE into squared-bias and variance components by Eq (25),

$$MSFE={\bar{\hat{e}}}^{2}+Var(\bar{\hat{e}})$$ (25)

where ${\bar{\hat{e}}}^{2}$ is the squared forecast bias, and $Var(\bar{\hat{e}})$ is variance of forecasting error. Another important statistic is the MSFE-adjusted of Clark et al. [95]. MSFE-adjust is a one-sided (upper-tail) measure to test the null hypothesis that the historical average MSFE is less than or equal to the forecast regression MSFE against the alternative hypothesis that the historical average MSFE is greater than the forecast regression MSFE. The MSFE-adjust statistic is obtained by the following calculation in Eq (26),

$$MSFE_{adj}={MSFE}_{a}-{MSFE}_{b}+\frac{\sum_{k=t+horizon+1}^{T}{\left(\hat{R_{k,a}}-\hat{R_{k,b}}\right)}^{2}}{T-t-horizon}$$ (26)

where *MSFE*_*a*_ and *MSFE*_*b*_ denote the MSFE statistic obtained by using predictive model and benchmark model, $\hat{R_{k,a}}$ and $\hat{R_{k,b}}$ mean the prediction value of Bitcoin asset by using prediction model and the benchmark model. For more details on these statistics, you may refer to Yin et al. [96].

In this paper, due to the length of our full sample, we set the length of the rolling window to 200 and the forecast horizon is set to 1, 2, and 4, respectively. Detailed results are shown in Table 11.

**Table 11:** Out-of-sample prediction results with different forecast horizons.

	VAR	Higher moment	Asymmetry	Interactive	OLS forecast with Dow	OLS forecast with Emu
Panel A: Bitcoin return						
a) Horizon = 1						
*R*^2^	0.0834	0.1184	0.0521	-0.0082	-0.1324	-0.0886
MSFE	0.0094	0.0090	0.0097	0.0103	0.0116	0.0111
${\bar{\hat{e}}}^{2}$	8.26*10^−6^	2.99*10^−6^	6.66*10^−6^	1.29*10^−5^	2.26*10^−6^	3.57*10^−5^
var($\bar{\hat{e}}$)	0.0094	0.0090	0.0097	0.0103	0.0116	0.0111
MSFE-adj	3.0683	1.9567	2.9660	2.4386	2.2317	1.5992
*P*	0.0011	0.0252	0.0015	0.0074	0.0128	0.0549
b) Horizon = 2						
*R*^2^	0.0863	0.1314	0.0542	-0.0238	-0.2433	-0.0786
MSFE	0.0092	0.0088	0.0096	0.0104	0.0126	0.0109
${\bar{\hat{e}}}^{2}$	1.54*10^−5^	1.23*10^−5^	1.23*10^−5^	1.34*10^−5^	7.58*10^−8^	4.86*10^−5^
var($\bar{\hat{e}}$)	0.0092	0.0088	0.0096	0.0103	0.0126	0.0109
MSFE-adj	3.0765	1.9674	2.8980	2.2834	2.2390	1.6352
*P*	0.0010	0.0246	0.0019	0.0112	0.0126	0.0510
c) Horizon = 4						
*R*^2^	0.1133	0.1513	0.0742	0.0269	-0.2378	-0.0374
MSFE	0.0090	0.0086	0.0094	0.0099	0.0126	0.0106
${\bar{\hat{e}}}^{2}$	9.92*10^−6^	1.03*10^−5^	4.13*10^−6^	1.46*10^−5^	1.04*10^−6^	2.35*10^−5^
var($\bar{\hat{e}}$)	0.0090	0.0086	0.0094	0.0099	0.0126	0.0105
MSFE-adj	3.4190	2.0829	3.0692	2.7863	2.3659	1.8998
*P*	3.14*10^−4^	0.0186	0.0011	0.0027	0.0090	0.0287
Panel B: Bitcoin realized volatility						
a) Horizon = 1						
*R*^2^	-0.0652	-0.1136	-	-0.2527	-0.4038	-0.3218
MSFE	0.0006	0.0006	-	0.0007	0.0008	0.0007
${\bar{\hat{e}}}^{2}$	2.78*10^−6^	5.94*10^−6^	-	5.21*10^−6^	1.24*10^−5^	3.16*10^−6^
var($\bar{\hat{e}}$)	0.0006	0.0006	-	0.0007	0.0008	0.0007
MSFE-adj	1.3989	1.3133	-	0.9559	0.1872	-0.0049
*P*	0.0809	0.0945	-	0.1696	0.4258	0.5020
b) Horizon = 2						
*R*^2^	-0.0422	-0.0741	-	-0.2236	-0.2495	-0.2520
MSFE	0.0006	0.0006	-	0.0007	0.0007	0.0007
${\bar{\hat{e}}}^{2}$	4.34*10^−6^	8.19*10^−6^	-	6.40*10^−6^	8.45*10^−6^	4.52*10^−6^
var($\bar{\hat{e}}$)	0.0006	0.0006	-	0.0007	0.0007	0.0007
MSFE-adj	1.3191	1.2281	-	0.8620	1.0241	0.0026
*P*	0.0936	0.1097	-	0.1943	0.1529	0.4990
c) Horizon = 4						
*R*^2^	-0.0526	-0.0689	-	-0.1800	-0.2439	-0.2484
MSFE	0.0006	0.0006	-	0.0007	0.0007	0.0007
${\bar{\hat{e}}}^{2}$	5.52*10^−6^	9.37*10^−6^	-	7.41*10^−6^	1.39*10^−5^	6.57*10^−6^
var($\bar{\hat{e}}$)	0.0006	0.0006	-	0.0007	0.0007	0.0007
MSFE-adj	1.2508	1.2720	-	1.1478	0.9424	0.0158
*P*	0.1055	0.1017	-	0.1255	0.1730	0.4937

Note: This table reports the results of out-of-sample forecasts with the rolling window sets to 200, while the forecast horizon equals to 1, 2, and 4, respectively.

Table 11 summarizes the out-of-sample prediction results of linear, i.e., VAR analysis, and non-linear models, i.e., higher moment, asymmetric analysis, interaction and joint impacts analysis, respectively. Based on the prediction results in Table 11, we can conclude that the basic VAR forecasting model, higher moment, as well as the asymmetric models outperform the benchmark historical average model in both short and long horizon regarding Bitcoin return forecasting, as the R^2^_oos_s are greater than 0.05, which is selected as a criterion value for R^2^_oos_. The R^2^_oos_ is positive if the interactive terms between investor attention and Bitcoin return are controlled. However, the value is too small to regard a significant improvement than the basic historical average forecasting model in the case if we set the horizon to 4. If the returns of related assets are controlled, the forecasting models perform poor as *R*^2^s are negative. For the realized volatility forecasting, none of these models performs better than the benchmark model, i.e., historical average, as the *R*^2^s are negative.

To sum up, the predictive models combined with investor attention can indeed apply to forecast the Bitcoin return both in linear and non-linear model specifications. Despite the investor attention shows an excellent explaining power for realized volatility regarding the in-sample analyzes, the novel variable performs poor in out-of-sample forecasting. These facts do add another empirical evidence to support Welch’s [82] view on in-sample and out-of-sample analysis.

## Economic value analysis based on Bitcoin asset allocation exercise

Inspired by Neely et al. [97] and Wang et al. [93], in this part, we implement an in-depth analysis on the economic significance of investor attention. Specifically, we measure the economic values of Bitcoin asset return forecasts for a risk-averse investor. As a final exercise, we collect and compute the LIBOR return rate, which can be interpreted as the risk-free rate of return that an investor is willing to accept rather than adopt the given risky portfolio.

Assume that the mean–variance investors optimally allocate assets across risk-free assets and Bitcoin asset incorporating risk aversion into the asset allocation decision. We utilize Sharpe ratio (SR) as the measurement for portfolio performance according to Wang et al. [93]. Specifically, when identifying the weight for the risky Bitcoin asset in the constructed portfolio, we need to maximize the following equation of utility for the portfolio,

$$U_{t}(r_{t})=E_{t}(w_{t}*r_{t}+r_{t,f})-0.5*\gamma *{var}_{t}(w_{t}*r_{t}+r_{t,f})$$ (27)

where *w*_*t*_ is the weight of risky Bitcoin asset in the portfolio, *r*_*t*_ is the Bitcoin asset return in excess of risk-free rate, and *γ* denotes the risk aversion degree. According to Wang et al. [93], maximizing *U*_*t*_(*r*_*t*_), the optimal weight of risky asset can be achieved by Eq (28),

$$w_{t}^{*}=\frac{1}{\gamma }*(\frac{\hat{r_{t+1}}}{\hat{\sigma_{t+1}^{2}}})$$ (28)

where $\hat{r_{t+1}}$ and $\hat{\sigma_{t+1}^{2}}$ represent the mean and volatility forecasts of Bitcoin excess returns. Obviously, the optimal weight depends on the risk aversion degree *γ*. A higher value of *γ* implies that risky asset receives lower weight in the portfolio. The portfolio return is shown in Eq (29).

$$R_{t+1}=w_{t}^{*}*r_{t+1}+r_{t+1,f}$$ (29)

In this section, two benchmark models are selected to explore and compare the economic values of investor attention in the Bitcoin market. The first one is based on Rapach et al., Marquering et al., Campbell et al., Welch et al. and Wachter et al. [92, 98–101], among others. These authors chose the historical average model as the benchmark model to forecast the return in the out-of-sample, and then construct certain portfolio based on the historical average forecasts in stock market. Thus, the historical average benchmark model is adopted; However, the historical average benchmark model is measuring the effectiveness of the general predictive models in relation to a naive approach, without discriminating the nuances of the choice of variables that intervene in the model. All the predictive models used in this paper are actually a modification of autoregressive model (AR). Therefore, another benchmark model of autoregressive model, which is also adopted by Wang et al. [93] to explore a similar issue in the finance area, is adopted to fill the potential deficiencies brought by the historical average benchmark model and illustrate the economic values of investor attention in the Bitcoin market more precisely. Specifically, in this paper, the AR(4) is adopted as another benchmark model.

In fact, the CAPM model is also a widely used model in forecasting and combining assets [102, 103]. However, the model is not selected as another benchmark model in this paper. The reason is as follows. When CAPM model is adopted, the parameter of β is surely needed. Due to data unavailability, the parameter of β for the Bitcoin market as well as the cryptocurrency market is not available. Thus, the CAPM is not selected.

In this part, we set the risk aversion parameter γ in Eq (27) to 3, 6 and 9. Furthermore, we add a restriction condition of transaction cost represented by the parameter bps, and set the cost parameter as 0, 10 and 20 basis points per transaction, respectively. For more details on economic value, refer to Neely et al. [97] and Wang et al. [93]. The related results of turnover and sharp ratio in out-of-sample portfolio are shown in the following Table 12.

**Table 12:** Comparisons of portfolio performance measures.

	*γ*	Indicator	Benchmark forecasting (historical average)	Benchmark forecasting (AR(4) model)	VAR forecasting	Asymmetrical forecasting	High moment forecasting
bps = 0	3	turnover	0.0327	0.4962	0.05137	0.5469	0.5668
		SR	0.0935	0.1619	0.1586	0.1774	0.1624
	6	turnover	0.0313	0.3128	0.3584	0.3851	0.3420
		SR	0.0935	0.1550	0.1619	0.1806	0.1593
	9	turnover	0.0244	0.2122	0.2541	0.2785	0.2363
		SR	0.0935	0.1511	0.1503	0.1712	0.1461
bps = 10	3	turnover	0.0327	0.4962	0.5137	0.5469	0.5668
		SR	0.0933	0.1599	0.1565	0.1752	0.1601
	6	turnover	0.0313	0.3128	0.3584	0.3851	0.3420
		SR	0.0931	0.1531	0.1598	0.1784	0.1573
	9	turnover	0.0244	0.2122	0.2541	0.2785	0.2363
		SR	0.0931	0.1492	0.1485	0.1691	0.1443
bps = 20	3	turnover	0.0327	0.4962	0.5137	0.5469	0.5668
		SR	0.0931	0.1580	0.1545	0.1731	0.1577
	6	turnover	0.0313	0.3128	0.3584	0.3851	0.3420
		SR	0.0928	0.1511	0.1578	0.1763	0.1552
	9	turnover	0.0244	0.2122	0.2541	0.2785	0.2363
		SR	0.0926	0.1472	0.1466	0.1670	0.1426

Note: This table reports the portfolio performance for a risk-averse investor who allocates assets between Bitcoin and risk-free asset. Here, we compare the different proportional transaction costs of 0, 10 and 20 basis points per transaction to assure the robustness. *γ* is the risk-aversion degree. In this section, based on the results of out-of-sample forecasting, three forecasting models are reported, i.e., VAR, higher moment and asymmetric model.

From Table 12, we can conclude that on the one hand, the predictive regression forecasts incorporating investor attention outperform the historical average forecast in terms of economic values. It obviously supports the results in Section 5. Thus, it is of great importance to pay attention to these predictive models when constructing portfolio with a historical average benchmark model; on the other hand, when constructing portfolio with AR(4) benchmark model, asymmetrical forecasting model outperforms other models including the AR benchmark model, which indicates that the predictive model surely bring economic values. Furthermore, the main results cannot be altered by the change of transaction cost, which means that our results are robust. To sum up, using investor attention to predict the return of Bitcoin asset can bring significant economic benefits based on allocation exercises and results do not vary across different benchmark models.

## Conclusion

In this paper, we focus on the relationships between Bitcoin market and the novel investor attention to fill the current potential research gap. First, we conduct the basic linear granger causality test and the corresponding VAR analysis. The results show that investor attention is indeed the granger cause of Bitcoin market both in return and realized volatility, and the shock from investor attention can last for several weeks in Bitcoin market. Second, we analyze the nonlinear connections between the two markets, i.e., high order moment, asymmetry, interactive term as well as joint impacts with other assets. The results show sophisticated non-linear relationships both for Bitcoin return and realized volatility. Third, we implement one basic and several longer-horizon out-of-sample forecasts to fully explore the relationships between investor attention and Bitcoin market, specifically for the forecasting abilities of investor attention. The results show that regarding the Bitcoin return, incorporating the investor attention surely improve the forecasting accuracy. However, predictive models with investor attention do not outperform the benchmark model regarding the Bitcoin realized volatility. Finally, we conduct asset allocation by constructing risky investment portfolios consist of Bitcoin asset and a risk-free asset to analyze the economic values of out-of-sample forecasts. The results show that compared with the historical average benchmark model and the AR benchmark model, the Bitcoin earnings forecasts based on investor attention have significant economic values as the Sharpe ratio is indeed increased. In summary, investor attention is a non-negligible factor in Bitcoin market.

However, there are some disadvantages regrading this paper. For example, in our empirical analysis, the OLS estimation methodology is mainly used to investigate the influence of investor attention. In the future, attempts to adopt other methods may be studied. In addition, this article mainly focuses on investor attention closely related to the Bitcoin market, investor attention on other markets, i.e., oil market or gold market, may also be important factors affecting the Bitcoin market. These defects deserve in-depth studies in the future.
